# Correlation between marginal bone loss around dental implants and various systemic diseases: a cross-sectional study

**DOI:** 10.1186/s40729-024-00566-7

**Published:** 2024-10-24

**Authors:** Alicia Carlos, Hassan Ziada, Neamat Hassan Abubakr

**Affiliations:** 1 DMD Student, School of Dental Medicine, University of Nevada, Las Vegas, NV USA; 2 Clinical Science Department, School of Dental Medicine, University of Nevada, Las Vegas, NV USA; 3 Biomedical Science Department, School of Dental Medicine, University of Nevada, MS 7415, Las Vegas, NV 89106 USA

**Keywords:** Hypertension, Hyperlipidemia, Diabetes mellitus, Dental implants, Marginal Bone Loss (MBL)

## Abstract

**Purpose:**

Diminished bone levels or the lack of osseointegration can lead to higher rates of failure of dental implants. The present study is aimed to evaluate the correlation between hypertension, diabetes mellitus and hyperlipidemia, on the marginal bone loss (MBL) surrounding dental implants among patients attending the University of Nevada, Las Vegas dental clinics.

**Methods:**

Clinical notes from patients at the University of Nevada, Las Vegas (UNLV) dental clinics were analyzed using AxiUm™ software. The study included patients with dental implants diagnosed with hypertension, diabetes mellitus, and hyperlipidemia who attended the UNLV School of Dental Medicine clinics from 2012 to 2022. Exclusions were made for patients with acquired immune deficiency syndrome and those with a limited number of radiographs. A search was conducted using keywords such as ‘systemic disease,’ ‘marginal bone loss,’ ‘dental implant,’ ‘high cholesterol,’ ‘hypertension,’ and ‘diabetes’ within the system.

**Results:**

Out of 1,310 potentially eligible patients, 57 fulfilled the inclusion criteria. The total number of evaluated implants was 165. 18% of the sample patients were 55 to 64 years of age, and 79% were 65 or above. 45.6% of patients reported having more than four systemic diseases and 67% of patients had four or more prescription medications. Patients diagnosed with hypertension (78.95%) or hyperlipidemia (73.68%) had the highest presence of marginal bone loss surrounding the dental implant(s) while those with diabetes (40.35%) had the least amount of MBL. Patients diagnosed with both hypertension and hyperlipidemia (29.82%) experienced the highest incidence of MBL around implants. The medications prescribed to combat these health issues, such as statins and antihypertensive, also showed the same trends and corresponded with a higher prevalence of MBL.

**Conclusions:**

Within the limitations of the present investigation, patients diagnosed with hyperlipidemia and hypertension were more likely to exhibit MBL surrounding dental implants.

## Background

Dental Implants are a widely utilized option for patients suffering from tooth loss that can help restore both function and esthetics. The long-term survival of dental implant therapy is reported widely in various studies [1]. A dental implant failure can occur in the early stages due to a lack of osseointegration or in the late stage due to peri-implantitis [2]. Marginal Bone Loss [MBL] was defined as loss, in an apical direction, of alveolar bone marginally adjacent to the dental implant, in relation to the marginal bone level initially detected after the implant was surgically placed [3, 4]. There is a bidirectional relationship between systemic and oral health, which may compromise the survival of the dental implant [5].

Hypertension is defined as a systolic value over 140 mmHg and/or a diastolic reading of over 90 mmHg and is considered the main cause of premature diseases and death in the world [6]. Blood pressure readings over time are valuable predictors of an individual’s risk of developing cardiovascular disease (CVD). Not only does it impose a risk of CVD, it can lead to comorbidities such as chronic renal failure and stroke. Bone abnormalities have also been linked to hypertension including a decrease in the regeneration, density, and quality of the alveolar bone due to impaired calcium metabolism and delayed healing [7].

Hyperlipidemia occurs as a result of abnormal lipid levels within the blood. This is characterized by increased levels of total cholesterol, triglycerides and low-density lipoproteins (LDL) as well as a decrease in the levels of “good cholesterol” known as high density lipoproteins (HDL) [8]. Currently, nearly 86 million adults in the United States 20 years and older have total cholesterol levels above 200 mg/dL [9]. A high level of cholesterol is a major controllable risk factor for other health complications including heart disease, myocardial infarction, and cerebrovascular accidents. Several studies have investigated the effects of hyperlipidemia on bone and have found that elevated amounts of plasma lipoproteins may increase the number of osteoclasts in the alveolar bone [10]. This phenomenon will lead to the inhibition of osteoblastic activity needed for osseointegration of the dental implant [11].

Diabetes mellitus is one of the most common chronic health issues within the world and is directly correlated with impaired wound healing due to an overactive immune response to pathogens. Inflammatory mediators (IL-1, IL-6, IL-8) and tumor necrosis factor (TNF-ɑ) associated with diabetes are released into the oral tissues. These factors can lead to increased inflammation and a reduction in collagen synthesis which ultimately affects formation of bone and the healing capability of oral tissues [12]. Hyperglycemic conditions over the long-term may degrade the vascularity within the oral cavity and that supplying the alveolar bone [13]. Just as in hyperlipidemia, the differentiation of osteoclasts is promoted and osteoblast formation is inhibited. Due to these risk factors, dental implants may not be considered on these individuals. While complications are evident among diabetic individuals, they have been seen to diminish in those with properly controlled diabetes mellitus [13]. In 2016, a systematic review narrated a significant delay in the osseointegration of dental implants in poorly controlled diabetic patients [14]. A recent meta-analysis indicated a direct association between hyperglycemia and the risk of peri‐implant diseases, and there is a high risk for MBL of dental implants for type 2 diabetes mellitus (T2DM) control patients [15]. It has been suggested that successful implants must present ≤ 2 mm of MBL during the first year after placement, followed by ≤ 0.2 mm per subsequent year [16]. It was indicated that there is an increased risk of implant failure if the MBL was 0.44 mm, at six months post-loading [17].

In 2018, Neves et al. concluded that rheumatologic and cardiovascular disorders are associated with an increased risk of peri-implant pathology [18]. A cross-sectional biochemical study comparing the inflammatory and lipid profiles of patients with and without peri-implantitis suggested that even healthy individuals with peri-implantitis exhibited a low-grade systemic inflammatory state, evidenced by elevated circulating white blood cell levels, as well as dyslipidemia, characterized by increased LDL cholesterol and total cholesterol levels [19].

A recent umbrella review emphasized the need for further studies to assess the long-term effects of cardiovascular disease, neurological disorders, and the use of certain medications on dental implant survival rates [20]. Another systematic review also highlighted that more research is required before drawing definitive conclusions about the association between cardiovascular disease and peri-implantitis, as the current body of literature contains too few studies to establish a clear link [21]. The present study aimed to evaluate marginal bone loss (MBL) around dental implants in patients with hypertension, hyperlipidemia, and diabetes mellitus attending the University of Nevada, Las Vegas (UNLV) dental clinics. The null hypothesis proposed that there would be no significant difference in MBL among patients with hypertension, hyperlipidemia, and diabetes.

## Materials and methods

### Study design and population

This study is a cross-sectional study of patients who attended the University of Nevada, Las Vegas dental clinics.

### Search strategy

**Fig. 1:**
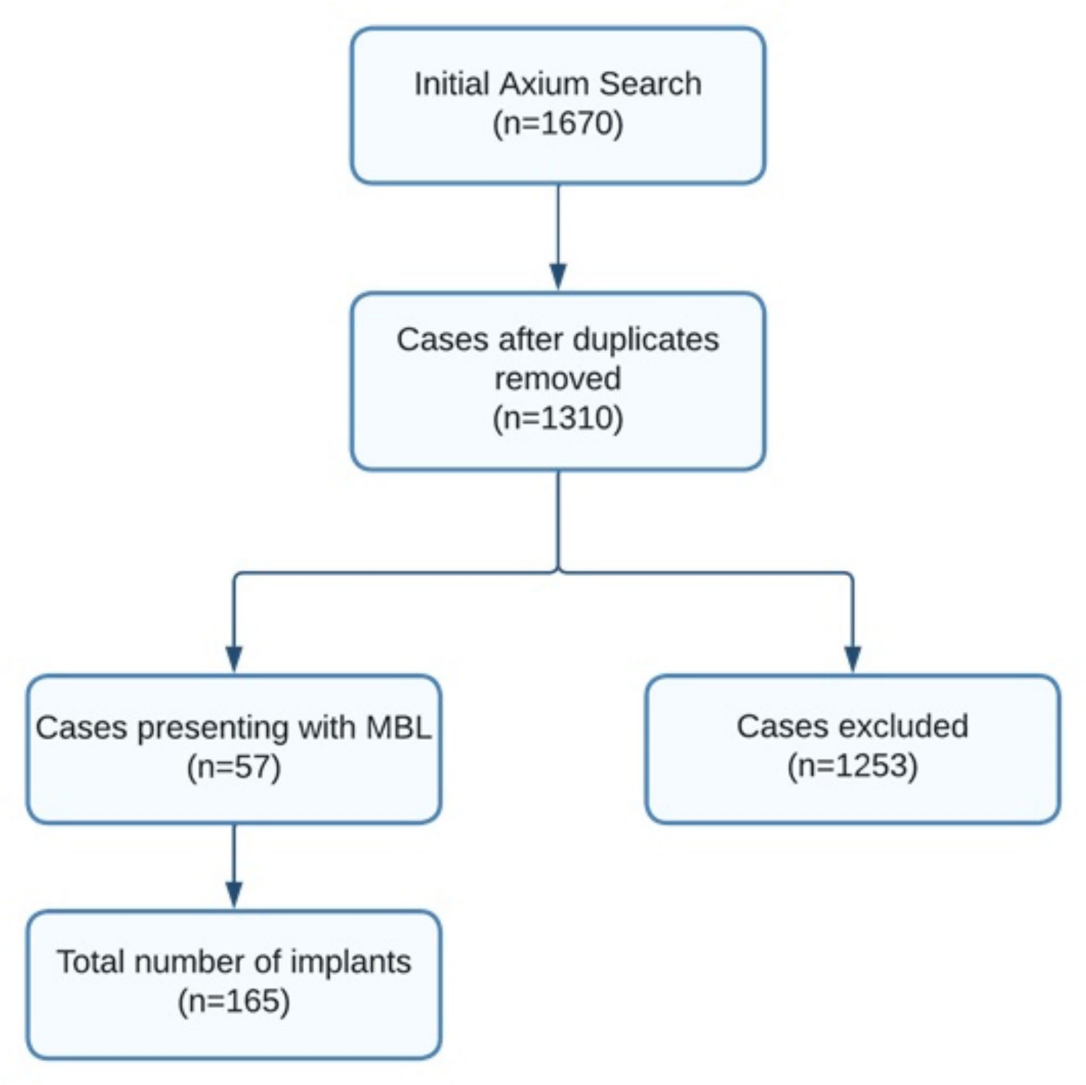
Flowchart depicting the search strategy to select eligible patients

Clinical notes from patients at the University of Nevada, Las Vegas (UNLV) dental clinics were analyzed using AxiUm™ software. AxiUm™ is a comprehensive dental practice management software for educational institutions, particularly dental schools. It helps streamline various administrative, clinical, and financial operations by integrating tools and features that manage patient records, treatment planning, billing, scheduling, and academic workflows. A search was conducted using keywords such as ‘systemic disease,’ ‘marginal bone loss,’ ‘dental implant,’ ‘high cholesterol,’ ‘hypertension,’ and ‘diabetes’ within the system (Fig. 1).

### Inclusion and exclusion criteria

The study included patients with dental implants diagnosed with hypertension, diabetes mellitus, and hyperlipidemia who attended the UNLV School of Dental Medicine clinics from 2012 to 2022. Exclusions were made for patients with acquired immune deficiency syndrome and those with a limited number of radiographs.

### Data extraction

All patients are informed during their intake to the UNLV dental clinics that they are being treated at an educational institution and their information may be used for research purposes. Their information is protected according to regulations dictated by the Federal Health Insurance Portability and Accountability Act of 1996 (HIPAA). Socio-demographic data including the patient’s age, gender, ethnicity, smoking habits (yes/no and type), and alcohol consumption (yes/no) were recorded. Dental and medical histories were collected from the existing chart records and evaluated. Data collected from these forms included systemic health issues and prescribed medications. Data were extracted by AC and NHA.

### Ethical approval

As no supplementary radiographs or examinations were conducted specifically for the study, and the data collected were analyzed and presented anonymously, the study was granted exempt status by the Institutional Review Board (IRB) of the University of Nevada at Las Vegas (UNLV; #UNLV-2022-256). The study adhered to the principles of good clinical practice in accordance with the World Medical Association (WMA) Declaration of Helsinki (1975), revised in 2013.

### Radiographic analysis

**Fig. 2:**
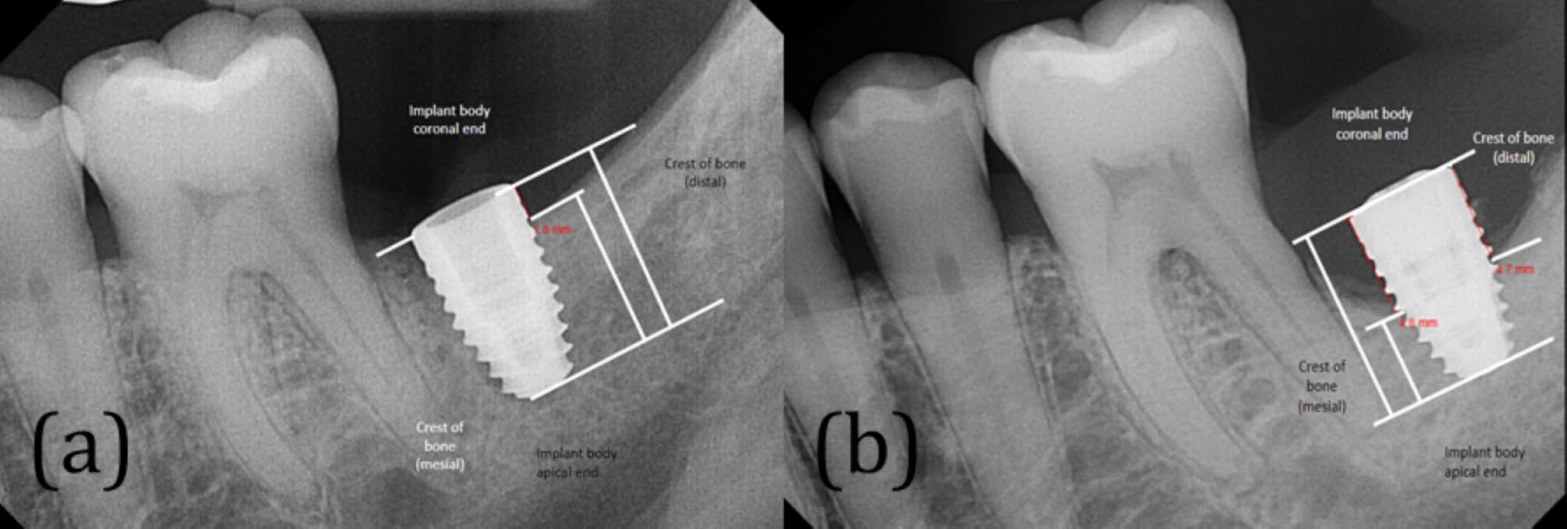
The method of measuring the marginal bone levels. **a** Marginal bone levels after implant placement; **b** marginal bone levels at the follow-ups (8 months after placement)

All radiographs were obtained using a long-cone technique at 70 kV and exposure time of 0.16 s. Only periapical radiographs were considered for the study, no CBCT imaging was included. All periapical radiographs were standardized using sensor holders and the parallel technique. Additionally, experienced dental technicians supervised all radiographic images taken at the radiology clinic to ensure quality and consistency. If the implant was placed at the UNLV dental clinic, peri-implant bone measurements were obtained from the most recent radiographs and radiographs at the time of placement. If the patient presented to the UNLV clinics with an existing implant, new radiographs taken over the course of at least 6 months were included in the data. The marginal bone level is defined as the vertical distance from the tip of the implant body to the coronal edge of the first bone-to-implant contact [4]. Marginal bone level was evaluated based on this principle as well as methods used in previous studies. Measurements were taken from the implant-abutment junction to the crest of the bone at both mesial and distal sides of each implant following the methodology described by Shi et al. [13] (Fig. 2). MBL was confirmed by comparing subsequent bone-to-implant contact levels to the initial radiographs. The MiPACS (Medical Imaging Picture Archiving and Communication System) is a comprehensive digital imaging system fully integrated with the axiUm™ dental software, and it was used for all radiographic measurements. MiPACS is the primary imaging module for capturing, viewing, and managing dental radiographs such as intraoral radiographs, panoramic images, and CBCT scans. MiPACS enhances clinical diagnostics with advanced image enhancement tools, including zoom, contrast adjustment, annotations, and measurement tools. One investigator (first author), conducted the measurement in order to eliminate inter-examiner variation. Using Cohen’s kappa in SPSS (version 28), the intra-examiner reliability was calculated to be 85%, indicating a strong level of agreement between repeated measurements.

## Results

After screening the 1,310 potentially eligible electronic Axium records, 57 patients with 165 implants met the selected inclusion criteria (Fig. 1). Table 1 shows the descriptive statistics of the sample. The majority of the patients were aged 65 and older (79%) followed by those between 55 and 64 years of age (18%). Social factors such as smoking and alcohol consumption accounted for < 50% of the sample (Table 1).

**Table 1:** Descriptive analysis of socio-demographic variables using Chi-Square Test

Variable	*N*%	x² value	*p*-value
*Gender*			
Male	47.37%	0.158	0.691
Female	52.63%		
*Age*			
18–24	0.00%	92.263	< 0.0001*
25–34	0.00%		
35–44	1.75%		
45–54	1.75%		
55–64	17.54%		
65 and >	78.95%		
*Alcohol*			
Yes	31.58%	7.143	0.008*
No	66.67%		
*Smoking*			
Yes	12.28%	32.439	< 0.001*
No	87.72%		

*p < 0.05; Significant

**Fig. 3:**
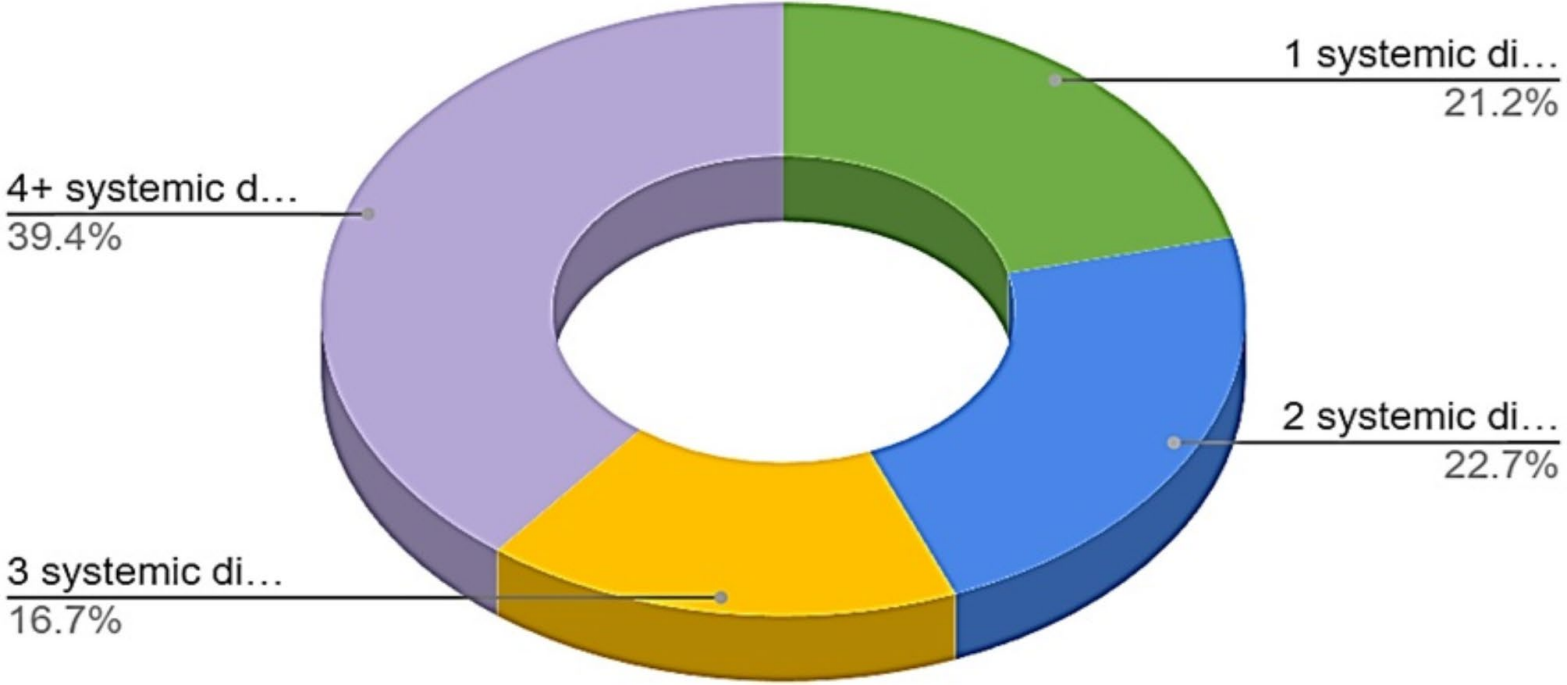
Distribution of systemic disease among MBL cases

The more systemic health disease a subject presented with, the more likely they had bone loss surrounding an implant as 45.6% of patients reported having more than four systemic diseases and 67% were taking four or more prescription medications (Fig. 3).

**Fig. 4:**
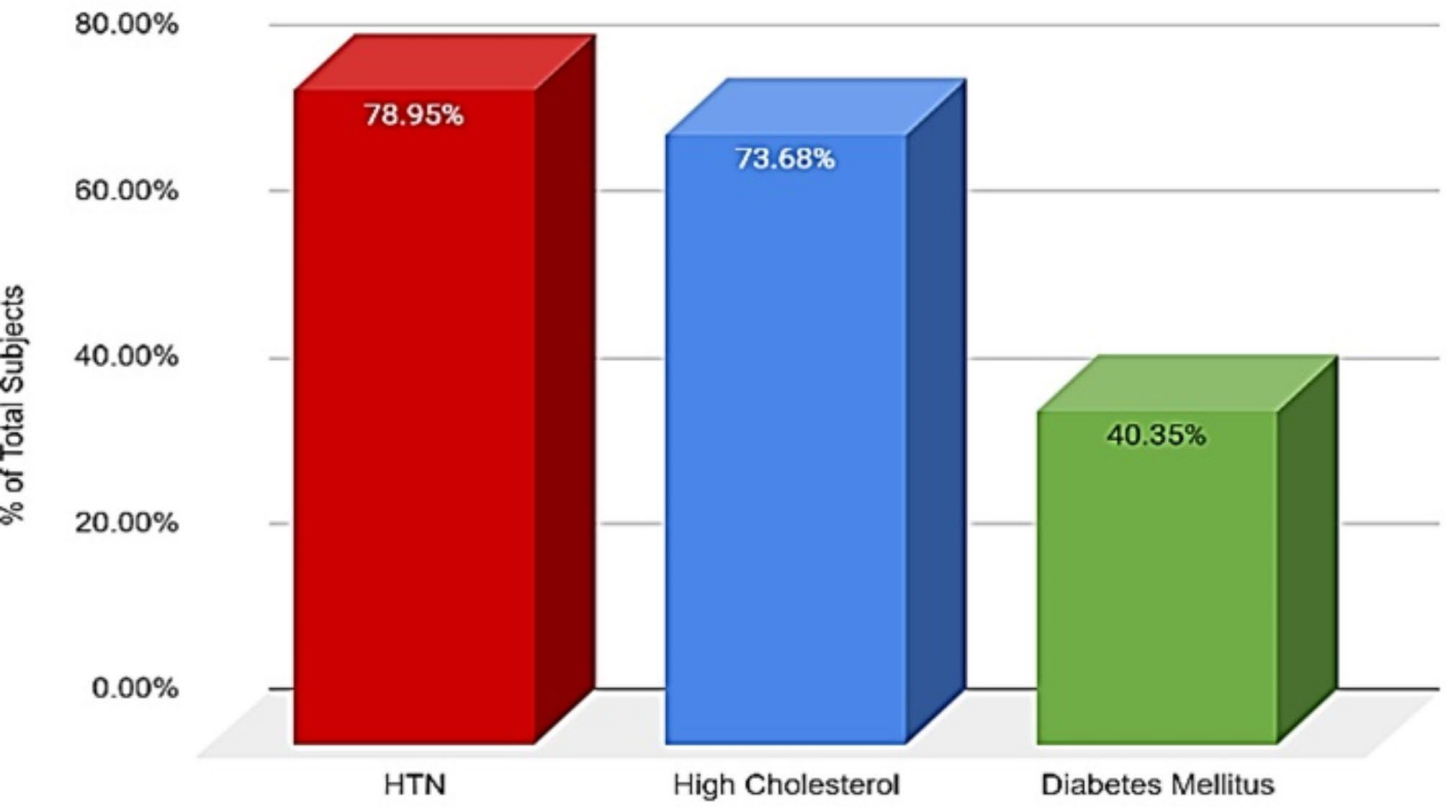
Systemic diseases with highest correlation to MBL (*HTN: Hypertension)*

A decrease in the marginal bone surrounding dental implants in this sample was strongly correlated with patients diagnosed with hypertension (78.95%) or hyperlipidemia (73.68%) compared to those with diabetes mellitus (40.35%). Patients diagnosed with both hypertension and hyperlipidemia comprised 29.82% of the sample data and held statistical significance (*p* < 0.05) (Fig. 4). Overall, varying combinations of these systemic diseases and comorbidities were more closely associated with peri-implant MBL than patients with a single systemic disease diagnosis.

Prescribed medications to combat these health issues, such as statins and antihypertensive, also showed the same trends and corresponded to a higher prevalence of MBL (Table 2). Over 90% of patients in each category reported taking the daily doses of medications on a regular basis.

**Table 2:** Analysis of systemic health and presence of MBL. Chi-Square Test: NS: *p* > 0.05; not significant; *p < 0.05; significant; **p < 0.001; highly significant

Variable	*N*	x² value	*p*-value
*Number of Medications*			
0–1 medications	4	33.107	< 0.001**
2 medications	7		
3 medications	7		
4 medications	10		
5 + medications	28		
*Medication Type*			
Diabetic medications	24	-	-
HTN medications	41	30.422	< 0.001**
Statin medications	40	34.381	< 0.001**
*# of systemic diseases*			
1 systemic disease	8	12.661	0.005*
2 systemic disease	14		
3 systemic disease	11		
4 + systemic disease	26		
*Systemic Disease*			
HTN	45	19.105	< 0.001**
HL	42	12.789	< 0.001**
DM	23	2.123	0.145; NS
*Comorbidities*			
HTN, HL	17	11.947	0.036*
HTN, DM	6		
HL, DM	4		
HTN, HL, DM	13		

[HTN: Hypertension; HL: hyperlipidemia; DM: DiabetesMellitus]

## Discussion

The aim of the present cross-sectional study was to investigate the correlation between hypertension, diabetes mellitus and hyperlipidemia, on the marginal bone loss (MBL) surrounding dental implants among patients attending the University of Nevada, Las Vegas dental clinics. After analyzing the results, it was found that there was a statistically significant difference in MBL between patients with cardiovascular diseases (HTN, hyperlipidemia) and diabetic patients. This study has successfully demonstrated that implant failure and peri-implant bone loss occur more in individuals that present with cardiovascular disease. Therefore, the null hypothesis was rejected.

Several studies have been published that report a direct relationship between diabetes and peri-implantitis. They concluded that diabetic patients, particularly those diagnosed with type 1, have a greater estimated MBL over time [22, 23]. This has been attributed to the reduced angiogenesis due to hypercoagulation as well as a hindrance of bone formation markers [24]. For Diabetic patients, it is important to note that the type and stability of diabetes is a determinant in the overall success of the implant. Deeper pocket depths, bleeding on probing and increased MBL were more commonly found among those with poor metabolic control compared to those with ‘well controlled’ diabetes [25]. This data correlates with the present study as diabetic patients presented with Type 2 diabetes had varying level of glycemic controls.

Bone metabolism is affected by hyperlipidemia through both osteoclasts and osteoblasts which may promote bone loss and inhibited osseointegration of dental implants [26]. In an experimental study on rats, Teken and Toker found that the rats that were fed a high cholesterol diet showed significantly lower bone-to-implant contact values than the control group [27]. The findings of their study give support to the hypothesis that hyperlipidemia can lead to a decrease in implant osseointegration and implant stability. Statin medications used to treat hyperlipidemia were also correlated to peri-implant bone loss. Behrami et al. conducted a retrospective study on the influence of statin use on the severity of peri-implantitis and the incidence of peri-implant bone loss [28]. However, several studies reported an increase in osseointegration of the implant body into the alveolar bone when coupled with Simvastatin. Implants were either coated with the Simvastatin or a gel containing the medication was placed into the alveolar socket at the time of placement [11, 29, 30]. These findings illustrate the need for further investigation into the effects of hyperlipidemia and statins on endosteal implants.

Hypertension has a positive association with moderate to severe periodontitis as bleeding on probing, CAL, and pocket depths were poorer on hypertensive patients [31]. Singh et al. found that hypertension led to a 20.8% increase in the failure of dental implants. This phenomenon may be due to impaired calcium metabolism and delayed healing associated with higher arterial pressure [32]. In a cohort study by Wu et al., of the 1,449 implants, a failure rate of 0.6% was observed in people using antihypertensive drugs and 4.1% for nonusers [33]. The results of these two studies show a possible failure of compliance in hypertensive patients taking their medications. This should be accounted for in the present study as the data relies on self-reporting from patients. Future studies should also evaluate the influence of time and follow-up periods on MBL which was not considered in this study.

There are several limitations to the present study. First, this is a cross-sectional dental-record based study that relied on the accuracy of the examination and documentation. Incomplete documentation was anticipated as patients are seen by a variety of students at University Dental Clinics. Second, there was a lack of quantifiable measurements of MBL radiographically and also no CBCTs were used in the investigation. The correlation was related to the main and mostly reported systemic diseases and not all systemic diseases. The measurements were performed as a visual representation of the effects associated with specific health issues and medications rather than a diagnostic tool. Third, oral hygiene and parafunctional habits could not be properly assessed due to the nature of this study; therefore the oral environment and the forces applied to the prosthesis might have an impact on the clinical outcomes. Lastly, a limited number of subjects, implants and restoration types were identified within the study.

## Conclusion

Within the limitations of the present investigation, patients diagnosed with hyperlipidemia and hypertension were more likely to exhibit MBL surrounding dental implants. Further investigation is required to correlate the presence of MBL around dental implants with antihypertensive and statin medication use.

## Data Availability

No datasets were generated or analysed during the current study.

## Abbreviations

MBL: Marginal Bone Loss
HTN: Hypertension
HL: Hyperlipidemia
DM: Diabetes mellitus
